# Precise Gene Modification Mediated by TALEN and Single-Stranded Oligodeoxynucleotides in Human Cells

**DOI:** 10.1371/journal.pone.0093575

**Published:** 2014-04-01

**Authors:** Xiaoling Wang, Yingjia Wang, He Huang, Buyuan Chen, Xinji Chen, Jianda Hu, Tammy Chang, Ren-Jang Lin, Jiing-Kuan Yee

**Affiliations:** 1 Department of Virology, Beckman Research Institute of City of Hope, Duarte, California, United States of America; 2 Department of Molecular and Cellular Biology, Beckman Research Institute of City of Hope, Duarte, California, United States of America; 3 Bone Marrow Transplantation Center, The First Affiliated Hospital, Zhejiang University, Hangzhou, Zhejiang, China; 4 Department of Hematology, Union Hospital of Fujian Medical University, Fuzhou, Fujian, China; Johns Hopkins Univ. School of Medicine, United States of America

## Abstract

The development of human embryonic stem cells (ESCs) and induced pluripotent stem cells (iPSCs) facilitates *in vitro* studies of human disease mechanisms, speeds up the process of drug screening, and raises the feasibility of using cell replacement therapy in clinics. However, the study of genotype-phenotype relationships in ESCs or iPSCs is hampered by the low efficiency of site-specific gene editing. Transcription activator-like effector nucleases (TALENs) spurred interest due to the ease of assembly, high efficiency and faithful gene targeting. In this study, we optimized the TALEN design to maximize its genomic cutting efficiency. We showed that using optimized TALENs in conjunction with single-strand oligodeoxynucleotide (ssODN) allowed efficient gene editing in human cells. Gene mutations and gene deletions for up to 7.8 kb can be accomplished at high efficiencies. We established human tumor cell lines and H9 ESC lines with homozygous deletion of the microRNA-21 (miR-21) gene and miR-9-2 gene. These cell lines provide a robust platform to dissect the roles these genes play during cell differentiation and tumorigenesis. We also observed that the endogenous homologous chromosome can serve as a donor template for gene editing. Overall, our studies demonstrate the versatility of using ssODN and TALEN to establish genetically modified cells for research and therapeutic application.

## Introduction

Developments in site-specific gene editing technologies such as zinc finger nucleases (ZFNs), transcription activator-like effector nucleases (TALENs) and clustered regularly interspaced short palindromic repeats (CRISPRs) have greatly facilitated disease modeling in animals and in pluripotent stem cells [1]–[8]. Among these technologies, CRISPRs have spurred great interest due to the ease of construction. However, recent findings about the off-target effects of CRISPRs confound their wide-spread application [9]-[11]. In contrast, TALENs exhibit high targeting specificity with little off-target effect except in one study [8], [12], [13]. Transcription activator-like effectors (TALEs) are important virulence factors first identified in plant pathogenic bacteria *Xanthomonas spp*.[5], [14], [15]. They directly bind to DNA via a central domain of tandem repeats and function as transcriptional activators [16], [17]. Each repeat consists of a 33- to 35- amino acid motif. The amino acid sequences of the repeats are nearly identical except for residues 12 and 13, the so-called repeat variable diresidues (RVD), which determine the DNA targeting specificity with one RVD targeting one nucleotide [18]. This relationship allows the engineering of specific DNA binding domains by assembling repeats with the appropriate RVDs. TALENs are derived from fusing the engineered TALE DNA binding domain to the Fok1 nuclease domain, which generates double-strand DNA breaks (DSBs) when two Fok1 nucleases dimerize [8], [19], [20].

Site-specific gene editing is based mainly on homology-directed recombination (HDR) between a gene locus and an exogenous DNA fragment. The efficiency of HDR in human cells is strongly stimulated by DSBs in the genome created by site-specific nucleases [21]. Non-homologous end joining (NHEJ) and HDR represent two major pathways for repairing DSB. NHEJ is error prone and can introduce mutations at the site of DSB to affect the expression of a protein coding-gene or microRNA as demonstrated in several model systems, including zebrafish, *Xenopus*, pig, mouse, and rat [4], [22]–[31]. In contrast, HDR is largely error-free and is typically accomplished with a sister chromatid, a homologous chromosome, or an exogenously provided donor template containing homology arms flanking the DSB [32]. However, construction of the donor template, the selection of clones with the desired gene modification, and subsequent removal of the selection marker constitute a lengthy process which unavoidably increases the stress and the risk of generating additional genome instability in human embryonic stem cells (ESCs) or induced pluripotent stem cells (iPSCs). Previous studies have demonstrated that single-strand oligodeoxynucleotide (ssODN) can be used as a template to generate point mutations and short sequence insertions in human cells and animal models [4], [22], [27]. In this study, we optimized the TALEN design to maximize its genomic cutting efficiency. We showed that using optimized TALENs in conjunction with ssODN as donor templates for HDR could mediate efficient gene editing in human cells. Gene mutations and gene deletions for up to 7.8 kb were accomplished at high efficiencies. Using this approach, we successfully established human tumor cell lines and H9 ESC lines with homozygous deletion of the microRNA-21 (miR-21) gene and miR-9-2 gene. These cell lines provide a robust platform to dissect the roles these genes play during cell differentiation and tumorigenesis. Our study also showed that homologous chromosome could serve as a donor template for gene editing. Taken together, our data demonstrates the versatility of using ssODN and TALEN to establish gene-edited human cell lines for research and therapeutic application.

## Materials and Methods

### Cell culture

H9 cells were obtained from National Stem Cell Bank (Madison, WI) and cultured on irradiated mouse embryonic fibroblasts (MEF) (GlobalStem, Inc., Rockville, Maryland). H9 cells were grown in ESC medium containing α-MEM/F12 supplemented with 20% knockout serum replacement, 0.1 mM nonessential amino acids, 0.1 mM 2-mercaptoethanol, and 1 mM L-glutamine. Basic fibroblast growth factor (bFGF) was added to a final concentration of 4 ng/ml prior to medium change. The culture medium was changed daily. HEK293T cells (CRL 3216, ATCC, Manassas,VA) were cultured in DMEM medium supplemented with 10% FBS, 2 mM L-glutamine, 100 U/ml penicillin and 100 mg/ml streptomycin. The K562 cell line (CCL 243, ATCC, Manassas,VA) was maintained in RPMI 1640 medium complemented with 10% FBS and 2 mM L-glutamine. The cells were cultured at 37°C in a humidified chamber with 5% CO_2_ in air, and passaged 1∶10 twice a week.

### TALEN design and construction

TAL Effector -Nucleotide Targeter 2.0 (https://tale-nt.cac.cornell.edu/) was used to find TALEN target sites [33]. Golden Gate TALEN and TAL Effector Kit from Addgene were used for TALEN repeats assembly [5].

### TALEN cutting efficiency evaluation

HEK293T cells were plated at 40% confluence in 48-well plates and were transfected with 0.2 μg of each TALEN plasmid using Lipofectamine 2000. Forty eight hours after transfection, genomic DNA was extracted with Epicentre QuickExtract solution (Epicentre Biotechnologies, Madison, WI). Approximately 8000 genome equivalents were used as input for PCR. TALEN activity was assayed via Surveyor nuclease following the manufacturer's protocol (Transgenomic, Omaha, NE). Image J was used to quantify the percent gene modification by measuring the intensity of bands separated by agarose gel post digestion. The following formula was used to calculate the percentage of gene modification: % gene modification  = 100×[1−(1−fraction cleaved)^1/2^]; % cutting efficiency (NHEJ)  = 100× sum of the cleavage product peak/(cleavage product+ parent peak). Primers used for monitoring gene modification at miR-9, miR-21, TAT intron 3, TAT exon 12 and SF3b1 locus were as follows: M9F2 (5′- tcctggacgaccactcttcggt-3′) and M9R2 (5′- gcagctgcaacaacccctctca-3′) for miR-9, TATF2 (5′-tggggacactactgaggggctg-3′) and TATR2 (5′- tcccgagacccggttcccaa-3′) for TAT intron 3, TATF3(5′-gcatcccagtcatgggagctgaat-3′) and TATR3 (5′-acctgcctggagagagcgtgt-3′), Surveyor L1 (5′-tggggttcgatcttaacagg-3′)and Surveyor R1 (5′- ctgcattgtgggttttgaaa-3′)for miR-21 and for SF3B1. The amplification was carried out with Hotstar Taq (Qiagen, Valencia, CA), using the following cycling conditions: 95°C for 15 min for initial denaturation; 35 cycles of 94°C for 30 s, 60°C for 30 s and 72°C for 30 s; and a final extension at 72°C for 5 min. Statistical significance was calculated using a paired Student's t test.

### ssODN mediated gene editing

The sequence of ssODN used in the TAT point mutation, 7.8 kb deletion, miR-21 and miR-9 gene modification study is as follows: 5′-ataatggctatgccccatccatcggtaagctcctcctgagacccatacctggatcctgccaaatctttagtgctcttataacaggactaaatgtctagc-3′, 5′-ataatggctatgccccat ccatcggtaagctcctcctgagactccatacctggatcctgccaaatctttagtgctcttataacaggactaaatgtctagc-3′, 5′- accatcgtgacatctccatggctgtaccaccttgtcggatcccagcatcattgtttataatcagaaactctggtccttct-3′, and 5′-aaggatcaggacctggagtctggcaagaggaagacagaggatccttcaagatcgccggggagcgtgtga gaatgaaagac-3′. 125 ng of each TALEN plasmid and 2.5 μl 1 μM ssODN were transfected into HEK293T cells using Lipofectamine 2000. Limiting dilution was performed at the following day. Ten days later, genomic DNA was extracted for genotyping using QuickExtract solution. TAT gene mutant clones were first screened with mutation-specific PCR primers: TATR2 & TATBAMF3 (5′-cctgagactccatacctggatc-3′). Genotypes were further confirmed by PCR with primers TATF2 and TATR2 followed by a *BamH*I digestion. The two expected DNA fragments were 274 bp and 140 bp. TAT, miR-21 and miR-9 deletion mutation clones were screened via deletion PCR assay with primersTATF2 and TATR3, Surveyor L1 and R1, M9F2 and M9R2. The amplification was carried out with JumpStart Taq (Sigma, St. Louis, MO), using the following cycling conditions: 94°C for 1 min for initial denaturation; 35 cycles of 94°C for 30 s, 58°C for 30 s and 72°C for 30 s; and a final extension at 72°C for 5 min. The wild-type band for miR-21 was 430 bp and the targeted deletion resulted in a 310 bp band. A heterozygous clone, #84 was transfected and screened using the methods described above. The homozygous mutation was confirmed by digesting the PCR product with *BamH*I, which gave rise to 170 and 140 bp fragments.

### Electroporation, isolation of targeted clonal cell population and neural differentiation

The K562 cells were suspended in RPMI 1640 without FBS or antibiotics, at a concentration of 10^7^ cells per ml. A volume of 0.4 ml was transferred to a sterile electroporation cuvette (Bio-Rad Gene Pulser cuvette, 0.4 cm), and kept at room temperature for 15 min in presence of 16 μg of pPB-c-GFP plamid to analyze the efficiency of transfection to analyze the efficiency of electroporation, or 20 μg each TALEN pair and 250 nM ssODN. Electroporation was performed with a Bio-Rad Gene Pulser Transfection Apparatus (350 V, 500 μF).

Before electroporation, H9 cells were grown in feeder-free adherent culture in chemically defined mTeSR1 (STEMCELL Technologies, Vancouver, Canada) on plates coated with Matrigel (BD Bioscience) for one generation. The cells were pretreated with 10 μM Rock Inhibitor for 2 hours and dissociated into a single cell suspension with 1 mg/ml Accutase (Invitrogen). Two million cells were mixed with 1 μg of each TALEN plasmid, 1.5 μg GFP plasmid and 2 μg ssODN, and then electroporated with program B-016 using Nucleofector (Lonza AG). Cells were cultured in mTeSR1 supplemented with 10 μM Rock Inhibitor for 48 hours and dissociated by Accutase. GFP-positive cells were collected by FACS (FACS Aria II; BD Biosciences) and replated on irradiated MEF feeder cells at 1000 cells per well. Ten days after sorting, single colonies were recovered. Half of each colony was manually picked and transferred into QuickExtract DNA extract solution (Epicentre). DNA extractions were done according to manufacturer's protocol. PCR screening was conducted as mentioned above. Neural differentiation was performed following the protocols of Hu et al [34].

### Real-time PCR

To test the expression of miR-21 and miR-9, total RNA was isolated using mirVana microRNA extraction kit (Life Technology, Carlsbad, CA). 0.5 μg of total RNA was used for reverse transcription (RT) with the miScript II RT kit (Qiagen, Hercules, CA). miScript primer assay and precursor assay were used for miR-21 and miR-9 quantification. Quantitative PCR was carried out with the miScript SYBR PCR kit (Qiagen, Hercules, CA). Haploid copy number variation in miR-9-2 locus was calculated as previously described [35]. qPCR was conducted in triplicate in a 20 ul reaction using the iQ SYBR Green Supermix and iQ5 multicolor real-time detection system (Biorad, Hercules, CA). Primers used were as follows: M9F5 (5′- ggaatcttaagcgcggcaag -3′) and M9R5 (5′- aacaactcgcttcccacaca-3′), M9F6 (5′-ggggagcgtgtgagaatgaa-3′) and M9R6 (5′-tttctctcatcccacctttaatca-3′). Wild type H9 cells were used as a calibrator sample and GAPDH was used as a reference gene with primers: GAPDHF1 (5′- gcaaggtcatccctgagctg -3′) and GAPDHR1(5′- ggcaggtttttctagacggc -3′). All reactions were run at 40 cycles using standard condition following manufacturer's protocol. Haploid copy number was calculated based on the observed Ct values: 2^-ΔΔCt^ = (1+E)^−ΔCtgene^/(1+E)^−ΔCtreferencegene^. E = efficiency of the PCR reaction (set at default value 0.95), ΔCt_gene_ = difference in the Ct value between test sample and calibrator sample. ΔCt_referencegene_ = difference in the Ct value between test sample and the calibrator sample for reference gene.

### Immunofluorescence

For immunofluorescence staining, cells were fixed in 4% paraformaldehyde for 10 min at room temperature. After PBS washing, cells were permeabilized with 0.3% Triton X-100 for 45 min at room temperature. After removal of the Triton X-100 solution, cells were washed with PBS and stained at 4°C overnight with primary antibodies at appropriate dilutions. Primary antibodies used include Tra-1-81 (1∶200, eBioscience, San Diego, CA), Tra-1-60 (1∶100, Stemgent, Cambridge, MA), Oct4 (1∶100, Stemgent, Cambridge, MA), Sox2 (1∶100, Neuromics, Minneapolis, MN), β III-tubulin (1∶2000, Covance, Princeton, NJ), HB9 (1∶10, Hybridoma Bank, Iowa City, Iowa). Cells were stained at room temperature with the secondary antibody for 2 hours at 1∶800 dilution, including Cy3-conjugated goat anti-rabbit IgG (Chemicon, Temecula, CA) or Cy3-conjugated rabbit anti-mouse IgG antibody (Millipore). Nuclei were stained with DAPI (Life Technology, Carlsbad, CA) at a concentration of 1 μg/ml.

## Results

### Optimization of TALEN design

**Figure 1:**
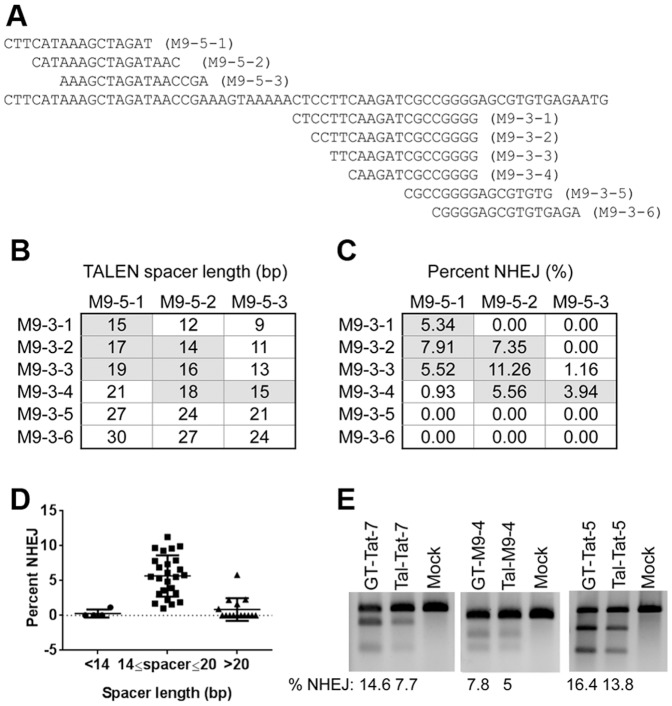
Optimization of TALEN design. (**A**) Target sequences of TALENs in the miR-9-2 locus. Target sequences for the three 5' TALENs and six 3' TALENs were shown. (**B**) Spacer lengths of the 18 TALEN pair combinations. (**C**) Percentage of NHEJ induced by the 18 TALEN pair combinations. Each TALEN pair shown in (**B**) was transfected into HEK293T cells and the Surveyor assay was carried out 72 h later. (**D**) Target cutting efficiencies of 40 TALEN pairs with various spacer lengths. Bar: average ± standard deviation, *p*<0.05, two tailed p value, student's T test. (**E**) Increased cutting efficiencies with the GoldyTALEN scaffold. Indicated TALEN pairs were transfected into HEK293T cells and NHEJ was measured by the Surveyor assay 72 hours later. GT: Goldy TALEN. Tal: wild-type TALEN.

We generated multiple TALEN pairs specific for different loci in the human genome (Table 1). Since a defined rule for TALEN design remains unclear, we arbitrarily searched for the TALEN target site preceded by a T, a feature identified within naturally occurring TALE recognition sites [36], [37]. We designed our TALENs with their target sites ranging between 15 and 30 bases (Table 1). The spacer between the two TALEN binding sites ranged from 15 to 29 bases. With the 22 TALEN pairs specific for different genes, we found that TALEN pairs with spacers larger than 20 bases generally were less efficient in cutting compared to those with smaller spacers (Table 1 and Figure S1). To exclude the possibility that the difference in cutting efficiency is due to the chromatin position effect, we designed three additional 5′ TALENs and six 3′ TALENs flanking the same cutting site in the miR-9-2 gene (Figure 1A). Cross matching individual TALENs generated 18 additional TALEN pairs with variable spacer lengths. We observed that TALEN pairs with spacer length of 14-20 bases were more effective than others (Figure1B and 1C). Together, data from these 40 TALEN pairs demonstrated that a spacer length of 14-20 bases was optimal for TALEN DNA cutting (Figure 1D). To further improve the TALEN cutting efficiency, we tested the GoldyTALEN scaffold which carried N- and C-terminal truncations of the native TALE protein [23]. Consistent with previous publications [7], [8], [22], [23], the GoldyTALEN scaffold enhanced gene cutting at the TAT and miR-9-2 loci relative to the native TALEN scaffold (Figure 1E). Our subsequent studies were therefore based on the use of the GoldyTALEN scaffold for TALEN construction.

**Table 1:** Summary of the design and cutting efficiency of TALEN pairs.

Gene	Name	Spacer (nt)	%NHEJ	TALEN-A sequence	TALEN-B sequence	
miR-9-2	M9-1	15	1	CCTGGACGACCACTCT	GCCAGACTCCAGGTCCTGATCCT	
	M9-2	29	2.29	ATCTAGCTGTATGAGT	CCCCGGCGATCTTGAAGGAGTTTTTACTTT	
	M9-3	22	5.82	CTTCATAAAGCTAGAT	CTCACACGCTCCCCGGCGATCTT	
	M9-4	19	9.6	CTTCATAAAGCTAGAT	CCCCGGCGATCTTGAA	X
	M9-5	16	3.24	GGCAAGAGGAAGACAG	AGATAACCAAAGATAA	
TAT	Tat-1	19	6.13	GTGAGCAGCACTACCAT	AGGAGTGTGATAAAT	
	Tat-2	16	5.78	CTGTGAGCAGCACTACCAT	AGGAGGAGTGTGATAAAT	
	Tat-3	22	0	GTGAGCAGCACTACCAT	AGTGTGATAAATAGGCCTGC	
	Tat-4	19	6.91	CTGTGAGCAGCACTACCAT	AGGAGTGTGATAAAT	
	Tat-5	16	9.95	GTGAGCAGCACTACCAT	AGGAGGAGTGTGATAAAT	X
	Tat-6	22	0	CTGTGAGCAGCACTACCAT	AGTGTGATAAATAGGCCTGC	
	Tat-7	18	4.85	CCTGAGACTCCATACCT	AGTGCTCTTATAACAGG	X
	Tat-8	29	0	GGCTATGCCCCATCCATCGGT	ACTGCCAAATCTTTAGTGCTCTTAT	
	Tat-9	21	0	CCATCGGTAAGCTCCT	ACTGCCAAATCTTTAGTGCTCTTAT	
miR-21	M21-1	16	3.57	CATGGCTGTACCACCT	AGACTGATGTTGACTG	
	M21-3	16	7.83	CCATATCCAATGTTCT	AGCATCATTGTTTAT	X
Sf3b1	Sf-1	19	2.24	AGTTAAAACCTGTGTTT	ATGAGCAGCAGAAAGTTCGG	
	Sf-2	23	1.64	ATTATCTGCTGACAGGCTAT	ATTTTGTTTAATGTGAACAT	
	Sf-3	23	0	AGGACAGCTGTCCTAAAAT	AAATGGAAAGGCATAGCTCT	
	Sf-4	15	1.87	ATGGTATCGAATCTT	AGCCTTTATGGAAGGGT	
	Sf-5	16	6.57	GTTTGGTTTTGTAGGT	AGAAAGTTCGGACC	
	Sf-6	16	9.67	AGTTAAAACCTGTGTT	GTGGATGAGCAGCAG	

TALENs were assembled according to the protocol described by Cermak *et al* [1]. The target sequences of each TALEN pairs (TALEN-A and -B) are listed. Cutting efficiency for each TALEN pair was measured using the Surveyor endonuclease. Percentage NHEJ is indicated. The pairs used for gene editing in this study are indicated with an “X” in the last column. TAT: tyrosine aminotransferase; Sf3b1: Splicing factor 3B subunit 1

### TALEN-mediated gene editing with ssODN in human cell lines

**Figure 2:**
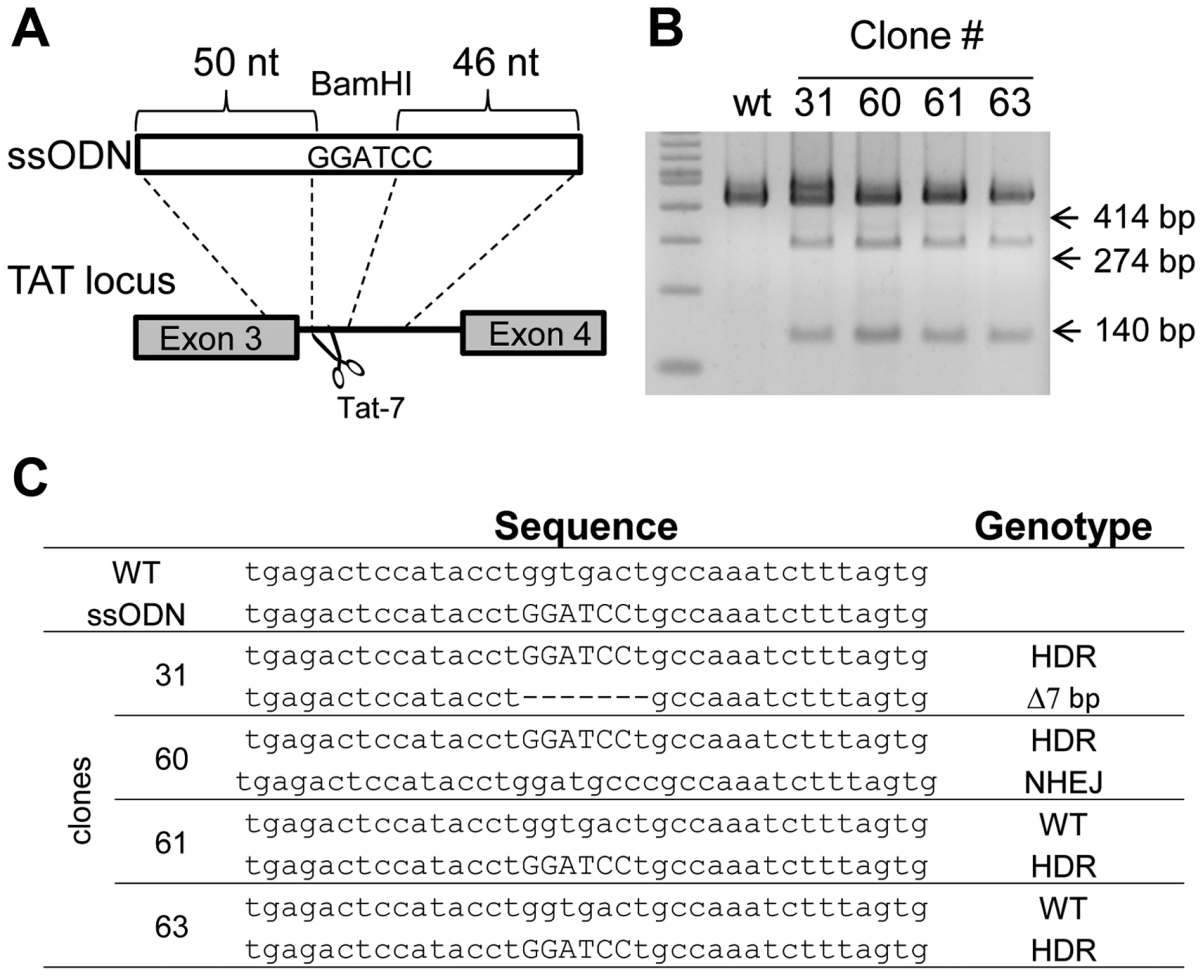
Induction of point mutations in the tyrosine aminotransferase (TAT) gene with ssODN and TALEN. (**A**) Schematic representation of the TALEN target region in the TAT gene and the ssODN used for HDR. (**B**) Analysis of randomly picked K562 clones transfected with the TALEN pair and ssODN. Individual clones were isolated by limiting dilution and subjected to genomic PCR using a primer pair flanking the TALEN cutting site. Gene edited clones were identified by BamH1 digestion that generated 274 and 140 bp fragments. (**C**) Sequence analysis of the TAT locus in the gene-edited clones. The PCR product from individual clones was TA cloned and subjected to sequencing analysis. HDR denotes ssODN-mediated homologous recombination. “Δ” denotes deletion. The deletion of the 7 bp in one of the TAT alleles in clone 31 is also shown.

We sought to use ssODN instead of a donor plasmid for gene editing to avoid the lengthy process of drug selection and subsequent removal of the selectable marker. We used a TALEN pair, Tat-7, which cut at intron 3 of the tyrosine aminotransferase (TAT) gene (Table 1 and Figure 2A). We synthesized a 99-base ssODN with a *BamH*I site flanked by 50- and 46-base homology arms corresponding to the sequences 5′ and 3′ of Tat-7 TALEN cutting site (Figure 2A). K562 cells were electroporated with the Tat-7 expression plasmids and the ssODN. PCR amplification of the TAT locus in individual clones followed by *BamH*I digestion showed that out of 150 randomly picked clones, 4 (2.7%) carried ssODN mediated HDR, which were further confirmed by sequencing (Figure 2B, 2C). This represents an underestimation of the homologous recombination efficiency since the transfection efficiency in K562 cells in around 50%. Our results suggest that ssODN is well-suited for generating point mutations at the DSB created by TALEN.

**Figure 3:**
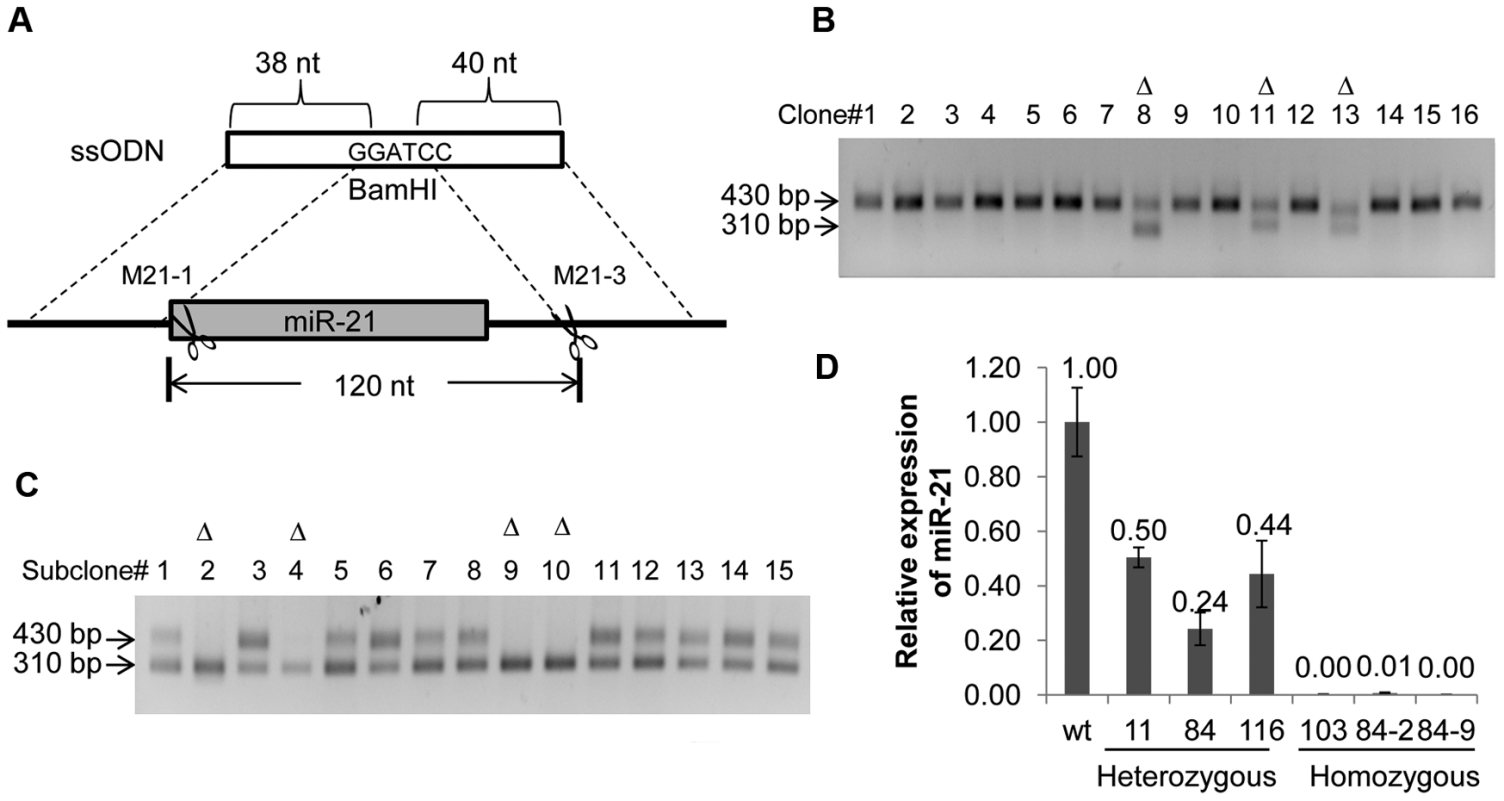
TALEN and ssODN-mediated deletion of the stem-loop structure in the miR-21 locus. (**A**) Schematic representation of the ssODN and its target in the miR-21 locus. The shaded box denotes the stem-loop structure of the miR-21 gene. The TALEN cutting site and the size of the ssODN-mediated deletion are indicated. (**B**) Representative picture of PCR screening of individual clones with gene editing in the miR-21 locus. HEK293T cells were transfected by the ssODN and M21-3. Gene edited clones were screened with a pair of PCR primer flanking the TALEN cutting site in the miR-21 gene. Positive clones exhibiting a 120-bp shorter PCR fragment are indicated by "Δ". (**C**) Representative picture of PCR screening of complete miR-21 knockout clones. Clone 84 was transfected by the ssODN and M21-1, and individual clones were screened as described in b. Clones with homozygous deletion exhibit only a single PCR fragment which is 120-bp shorter than the wild-type fragment. Potential miR-21 homozygous knockouts are indicated by "Δ". (**D**) Analysis of miR-21 expression in gene edited HEK293T clones. Total RNA from the clones indicated was isolated and subjected to qRT-PCR to assess the level of the mature miR-21 relative to that from parental HEK293T cells (wt). *p*<0.05.

Generating deletion is of great interest for loss-of-function studies. To determine whether ssODN can mediate small gene deletions, a *BamH*I-containing ssODN sharing 38- and 40-base sequence homology flanking the stem-loop structure of the miR-21 gene was synthesized (Figure 3A). Successful gene editing with the ssODN is expected to create a 120-bp deletion that removes the entire stem-loop structure of the miR-21gene and silences its expression. The M21-3 TALEN expression plasmids and the ssODN were co-transfected into HEK293T cells and PCR analysis of individual clones showed that 13 out of 110 clones (11.8%) exhibited a PCR product with reduced fragment size (Figure 3B). Sequence analysis confirmed the expected deletion in these clones with clone 103 containing a triallelic knockout of the miR-21 gene (Table 2).

**Table 2:** Sequence analysis of selected miR-21 clones used for quantitative RT-PCR (qRT-PCR) studies.

Clone No.	Sequence	Genotype
	ACATCTCCATGGCTGTACCACCTTGTCGG. 106 nt.CATTTAAACATTACCCAGCATCA	wild type
	ACATCTCCATGGCTGTACCACCTTGTCG GATCCCAGCATCA	ssODN
	**Selected heterozygous clones**	
11	ACATCTCCATGGCTGTACCACCTTGTCGGATCCCAGCATCATTGT	HDR
	ACATCTCCATGGCTGTACCACCTTGTCGG. 94 nt. CATTTAAACATTACCCAGCATCA	Δ 12 bp
	ACATCTCCATGGCTGTACCACCTTGTCGG. 106 nt.CATTTAAACATTACCCAGCATCA	WT
84	ACATCTCCATGGCTGTACCACCTTGTCGGATCCCAGCATCATTGT	HDR
	ACATCTCCATGGCTGTACCACCTTGTCGG. 106 nt. CAT-----------------CA	Δ 18 bp
116	ACATCTCCATGGCTGTACCACCTTGTCGGATCCCAGCATCATTGT	HDR
	ACATCTCCATGGCTGTACCACCTTGTCGG. 106 nt.CATTTAA---TTACCCAGCATCA	Δ 3 bp
	**Knockout clones**	
103	ACATCTCCATGGCTGTACCACCTTGTCGGATCCCAGCATCATTGT	HDR
	ACA-----------------------------//-------------------CAGCATCA	Δ 150 bp
84-2	ACATCTCCATGGCTGTACCACCTTGTCGGATCCCAGCATCATTGT	HDR
84-4	ACATCTCCATGGCTGTACCACCTTGTCGGATCCCAGCATCATTGT	HDR
84-9	ACATCTCCATGGCTGTACCACCTTGTCGGATCCCAGCATCATTGT	HDR
84-10	ACATCTCCATGGCTGTACCACCTTGTCGGATCCCAGCATCATTGT	HDR
84-27	ACATCTCCATGGCTGTACCACCTTGTCGGATCCCAGCATCATTGT	HDR
84-48	ACATCTCCATGGCTGTACCACCTTGTCGGATCCCAGCATCATTGT	HDR

Genomic PCR of individual miR-21 clones was carried out and the PCR products were TA cloned and sequenced. The sequence of the ssODN and wild-type cells is shown on top of the table. HDR denotes homology directed recombination. “Δ” denotes deletion which is indicated by a dished line.

To obtain more homozygous miR-21 knockout clones, we chose clone 84 to carry out a second round of gene knockout. This clone had one allele derived from ssODN-mediated HDR and the other derived from a NHEJ-mediated 18-bp deletion (Table 2). Since the 18-bp deletion abolished the binding of the original TALEN pair M21-3, we adopted a different TALEN pair, M21-1, for knocking out the remaining allele (Figure 3A). We identified 6 homozygous knockout clones out of 60 clones screened (10%) (Table 2 and Figure 3C). The level of miR-21 in these clones was determined by RT-PCR and the result showed that miR-21 expression was reduced in the heterozygous clones and completely silent in the homozygous knockout clones (Figure 3D), confirming the complete knockout of all three miR-21 alleles from HEK293T cells.

### A TALEN-mediated large genomic deletion in the TAT locus

**Figure 4:**
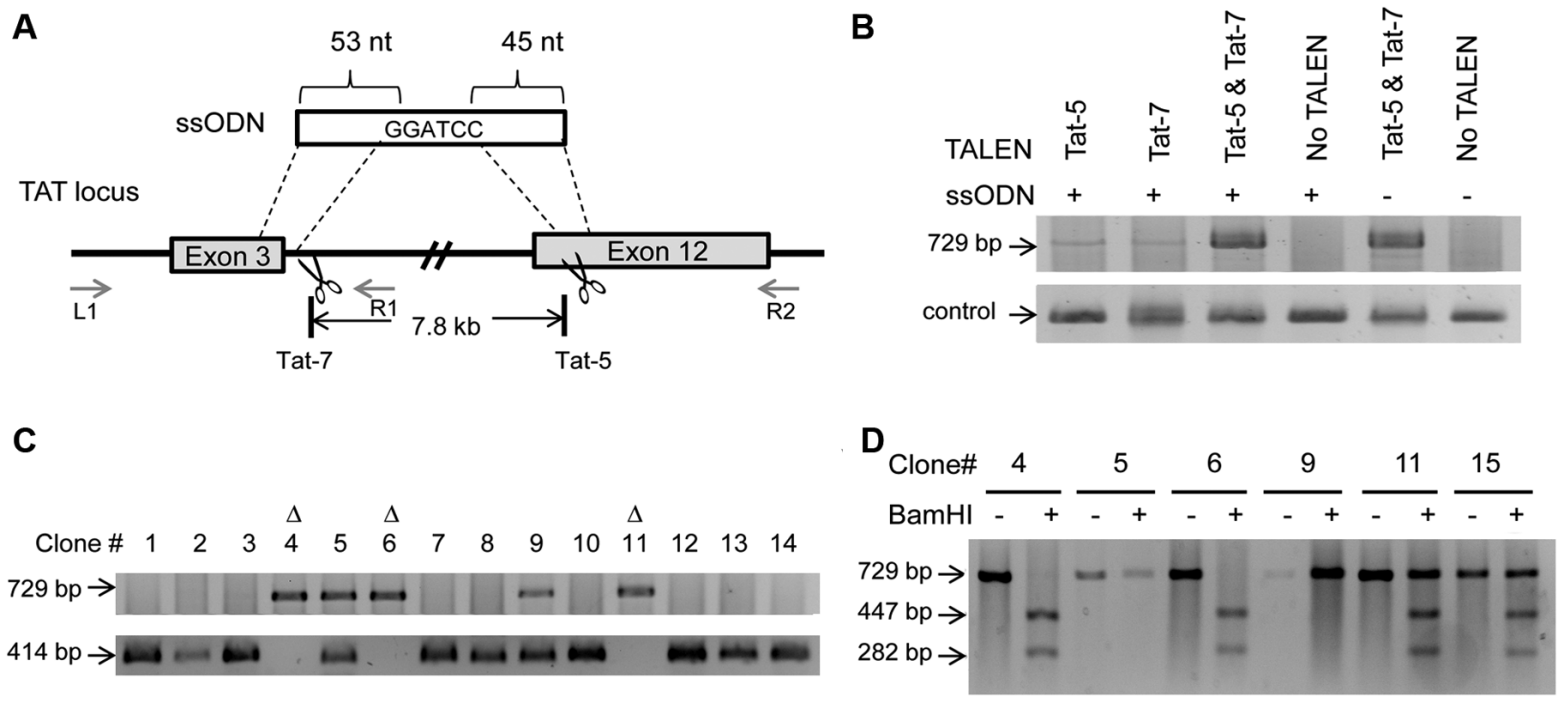
Deletion of a large genomic fragment with two TALEN pairs and an ssODN. (**A**) Schematic representation of the TALEN target regions in the TAT gene. Tat-7 and Tat-5 cut in intron 3 and exon 12, respectively, resulting in the deletion of a 7.8 kb genomic fragment. A100-mer ssODN used for HDR and the homology regions between the ssODN and the TAT gene are shown. The primers used for PCR screening of isolated clones, L1, R1 and R2, and their positions in the TAT gene are indicated. (**B**) TALEN and ssODN mediated a large genomic deletion in the TAT gene. HEK293T cells were transiently transfected with the TALEN and the ssODN as indicated, and the total genomic DNA harvested was subjected to PCR analysis using primer L1 and R2. A primer pair specific for the miR-9-2 gene was used for PCR as the loading control. (**C**) Representative picture of PCR analysis of individual HEK293T clones transfected with the two TALEN pairs and the ssODN. Top panel: targeted deletion in the TAT gene was amplified with L1 and R2, resulting in a 729 bp PCR fragment. Bottom panel: wild-type TAT locus was amplified with L1 and R1, resulting in a 414 bp PCR fragment. Homozygous knockout clones were indicated by “Δ”. (**D**) Validation of HEK293T clones containing HDR-mediated deletion. The PCR product from HDR-mediated deletion was digested by BamHI into two fragments with sizes of 447 bp and 282 bp.

Introduction of a small deletion or frame-shift mutation may not be sufficient to completely inactivate the gene function since RNA splicing or alternate transcription start sites can skip the deletion or mutation and produce a protein with partial function. Deletion of more than two critical exons or even the entire gene may be required to completely abolish the gene function. To evaluate whether ssODN could induce large genomic deletions, we synthesized a *BamH*I-containing ssODN that shared sequence homology with intron 3 and exon 12 of the TAT gene (Figure 4A). HDR mediated by this ssODN was expected to remove a 7.8-kb genomic fragment spanning the majority of the TAT gene. Initially, we screened individual HEK293T clones transfected by the TALEN pair Tat-7 and the ssODN with primer pair L1/R2 located outside of the deleted region (Figure 4A). We found that none of the 96 clones screened showed the expected deletion (data not shown). We then sought to test whether applying two TALEN pairs, Tat-5 and Tat-7 (Figure 4A), could cooperatively induce the deletion. HEK293T cells were transiently transfected with the TALEN pairs and the ssODN. PCR was used to semi-quantify the overall deletion events in pooled cells. We observed that deletions induced by a single TALEN pair and the ssODN were detectable but the signal was weak (Figure 4B). Cells treated with the two TALEN pairs exhibited a much stronger PCR band both with and without the ssODN (Figure 4B). Screening individual clones showed that 24 out of 59 clones (40.7%) transfected by the two TALEN pairs and the ssODN exhibited a 729-bp PCR fragment expected from the deletion of the 7.8 kb genomic fragment (Figure 4C, upper panel). Out of these 24 positive clones, the PCR product from 12 clones (20.4%) was cut by *BamH*I into the expected 447- and 282-bp fragments (Figure 4D), indicating the presence of at least one HDR-derived allele in these clones. The other 12 clones that could not be cut by *BamH*I were most likely derived from NHEJ (Figure 4C and D). These observations were further confirmed by cloning and sequencing the PCR products (data not shown). We then used L1 and R1, a primer positioned within the deleted region, to screen for the presence of the wild-type allele among these clones. Out of the 12 clones with HDR-mediated deletion, 3 clones (5.1%) failed to show the expected 414 bp wild-type band (Figure 4C, lower panel), suggesting homozygous deletion of the TAT gene in these clones. Thus, simultaneous administration of two TALEN pairs can efficiently remove a large genomic sequence between the two TALEN target sites and rejoin the genomic ends together. Although generating two distant cleavages in cis without the ssODN seems sufficient to create a relatively large deletion in the genome (Figure 4B), the presence of the ssODN can mediate the deletion precisely at the single nucleotide level.

### TALEN-mediated deletion of the miR-9-2 gene in the human H9 ESC line

**Figure 5:**
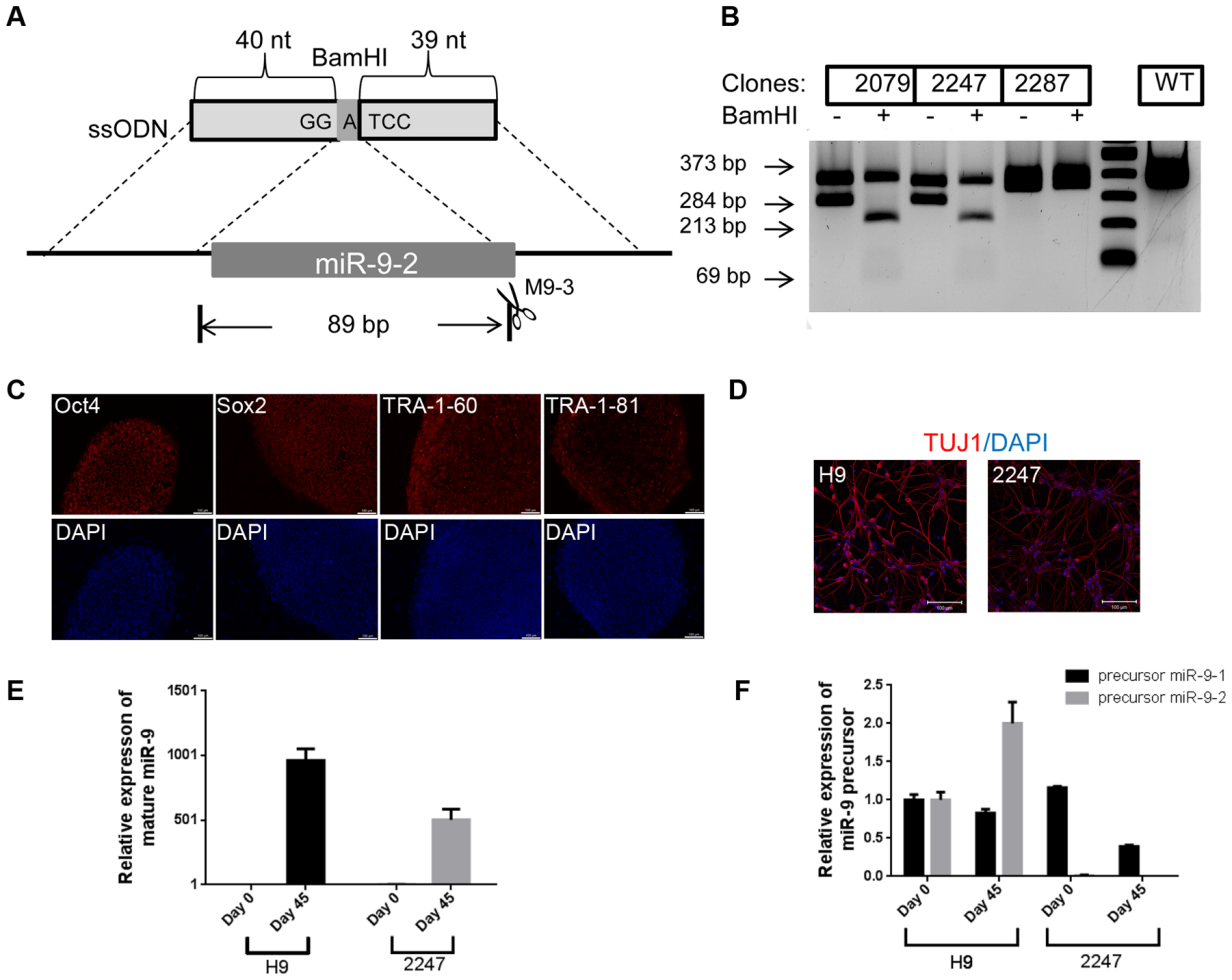
Knockout of the miR-9-2 gene in H9 cells. (**A**) Schematic representation of the miR-9-2 gene and a 100-mer ssODN used for miR-9-2 deletion. The box in the miR-9-2 locus indicates the stem-loop structure of the gene. Homology regions between the ssODN and the miR-9-2 gene are indicated. (**B**) Genomic PCR screening of the miR-9-2 gene deletion. H9 clones transfected with the TALEN and ssODN were isolated. Genomic PCR to amplify the region surrounding the TALEN cutting site followed by BamH1 digestion was carried out. The expected fragment sizes for wild-type and ssODN-mediated HDR are 373 bp and 284 bp, respectively. BamHI cuts the 284 bp fragment into 213 bp and 69 bp fragments. (**C**) Immunofluorescence staining of stem cell markers in undifferentiated clone 2247 cells. Nuclei were counterstained by DAPI. Scale bar = 100 μm. (**D**) Immunofluorescence staining of TUJ1 in neuronal cells derived from differentiated H9 and clone 2247 cells. Scale bar = 100 μm. (**E**) Level of mature miR-9 in H9 and clone 2247. Quantitative RT-PCR was used to measure the level of mature miR-9. Day 0 denotes the undifferentiated cells whereas day 45 denotes neuronal cells. *p*<0.05. (**F**) Level of precursor miR-9-1 and miR-9-2 in H9 and clone 2247. Pre-miR-9-2 was undetectable in clone 2247.

We next tested TALEN and ssODN mediated gene deletion in human ESC line. MiR-9 regulates neurogenesis through its action on the proliferation, migration and differentiation of neural progenitor cells [38]-[43]. Three genes, miR-9-1, miR-9-2 and miR-9-3 located on chromosome 1, 5, 15, respectively, encode miR-9 [44]. Although mature miR-9 from the three genes is identical in sequence, the precursor RNA sequences are different. It was reported that the mature miR-9 in human ESC-derived neuroprogenitors was mostly expressed from the miR-9-2 gene [40]. To determine the role miR-9-2 plays in ESC neuronal differentiation, we designed an ssODN that shared 40 bp and 39 bp sequence homology flanking the stem-loop structure of miR-9-2 gene (Figure 5A). HDR mediated by this ssODN was expected to delete the entire stem-loop structure of the miR-9-2 gene and silence its expression. We first tested this ssODN by co-transfecting HEK293T cells with a TALEN pair, M9-3 (Figure 5A), and the ssODN followed by random clone isolation. Out of 96 clones screened, 5 clones (5.2%) carried the expected 89-bp deletion mediated by HDR (data not shown). We then repeated the same procedure in H9 cells with a GFP expression plasmid included in the nucleofection mix to enrich for the transfected cells via cell sorting. To estimate the overall gene modification rate, we sequenced the miR-9-2 locus in 90 randomly picked H9 clones (clone 2101-2190). We found that 15 clones (16.7%) contained NHEJ-mediated mutations in at least one miR-9-2 allele and 5 of them contained mutations in both alleles (Table S1), suggesting efficient cutting by M9-3 in H9 cells. Based on genomic PCR screening, we identified two clones (clones 2079 and 2247) out of 300 clones (0.67%) carrying HDR-mediated deletion, which was further confirmed by *BamH*I digestion of the PCR product and sequencing (Figure 5B and Table S2). These clones not only retained their self-renewal capacity, but also maintained the ability to differentiate into neurons (Figure 5C, D). In addition to the expected ssODN-mediated deletion, clone 2247 had a NHEJ-mediated 13-bp deletion in one allele (Table S2). This deletion removed the complementary strand of the entire miR-9 seed sequence and presumably would disrupt proper miR-9-2 processing and impair its expression. We measured the expression of mature miR-9 in H9 and clone 2247 before and after neuronal differentiation. As expected, the level of mature miR-9 remained extremely low both in undifferentiated H9 cells and clone 2247 (Figure 5E, Day 0) [40]. Upon differentiation, the level of mature miR-9 was elevated to more than 1000 fold in H9 cells and approximately 500 fold in clone 2247 (Figure 5E, Day 45). We also measured the expression of the three miR-9 precursors in H9 and clone 2247 using quantitative RT-PCR. As reported previously [44], [45], our data showed that pre-miR-9-1 and pre-miR-9-2 were more abundant, whereas pre-miR-9-3 was barely detectable (data not shown). In clone 2247, pre-miR-9-2 expression was undetectable regardless of the differentiation status, consistent with the complete knockout of the miR-9-2 gene (Figure 5F). Based on these results, we concluded that the 13-bp deletion in miR-9-2 had a negative impact on its processing which contributed to the complete absence of miR-9-2 expression in this clone.

Since a homologous chromosome can act as the repair template in a substantial proportion of DSB repair events [46], [47], it was of interest to determine whether it could also facilitate the generation of homozygous gene knockout in ESCs. Clone 2287 represents an ideal candidate to test homologous chromosome-mediated HDR as this clone has one wild-type miR-9-2 allele and a 35-bp deletion in the other allele which was generated by NHEJ (Table S2). The 35-bp deletion eliminates the TALEN binding sites and renders the wild-type allele the only target for TALEN cutting. If homologous chromosome-mediated HDR occurred, we would expect to isolate homozygous knockout clones with the 35-bp deletion in both of the miR-9-2 alleles. To test this hypothesis, we co-transfected M9-3 and the ssODN into clone 2287. Out of 196 clones screened, 8 clones (4.1%) no longer exhibited a wild-type PCR band (Figure S2B). Among them, 3 clones (1.5%) had the expected ssODN-mediated 89-bp deletion of the wild-type allele, and one clones (0.5%), clone 2287-19, had a 35-bp deletion in the remaining wild-type allele (Figure S2B). This was verified by using PCR to measure the genomic copy near the region of the TALEN cutting site and direct DNA sequencing of the PCR product (Table 3 and Figure S2C). This result therefore suggested that the DSB generated by TALEN in the remaining wild-type miR-9-2 allele was repaired using the homologous chromosome with the 35-bp deletion as a template. The wild-type alleles in the remaining 4 clones were repaired by NHEJ as they showed deletions with variable sizes (Table 3 and Figure S2A). Based on this result, we were able to assess the total HDR efficiency at around 2% (4/196 clones) in H9 cells. We also measured the levels of miR-9 and pre-miR-9-2 in clone 2287-derived homozygous knockout clones and confirmed the complete absence of miR-9-2 expression in those clones (data not shown).

**Table 3:** Sequence analysis of miR-9-2 locus in subclones derived from clone 2287.

Clone No.	Sequence	Genotype
	ACAGAGG ATCCTTCAAGATCGCCGG	ssODN
Parental clone 2287	ACAGAGG.65 bp. GCTAGATAACCGAAAGTAAAAACTCCTTCAAGATCGCCGG	WT
	ACAGAGG.55 bp. -------------------------CTTCAAGATCGCCGG	Δ 35 bp
2287–114	ACAGAGGaTCCTTCAAGATCGCCGG	HDR
	ACAGAGG.55 bp. -------------------------CTTCAAGATCGCCGG	Δ 35 bp
2287–182	ACAGAGGaTCCTTCAAGATCGCCGG	HDR
	ACAGAGG.55 bp. -------------------------CTTCAAGATCGCCGG	Δ 35 bp
2287–191	ACAGAGGaTCCTTCAAGATCGCCGG	HDR
	ACAGAGG.55 bp. -------------------------CTTCAAGATCGCCGG	Δ 35 bp
2287–19	ACAGAGG.55 bp. -------------------------CTTCAAGATCGCCGG	Δ 35 bp
2287–151	ACAGAGG.55 bp. -------------------------CTTCAAGATCGCCGG	Δ 35 bp
	-------------------------------AAAAACTCCTTCAAGATCGCCGG	Δ 235 bp
2287–50	ACAGAGG.55 bp. -------------------------CTTCAAGATCGCCGG	Δ 35 bp
	ACAGAGG.65 bp. GCTAGA--------------------TTCAAGATCGCCGG	Δ 20 bp
2287–68	ACAGAGG.55 bp. -------------------------CTTCAAGATCGCCGG	Δ 35 bp
	ACAGAGG.62 bp. -------------------AAACTCCTTCAAGATCGCCGG	Δ 22 bp
2287–72	ACAGAGG.55 bp. -------------------------CTTCAAGATCGCCGG	Δ 35 bp
	ACAGAGG.65 bp. GCTAGAT-----------AAAACTCCTTCAAGATCGCCGG	Δ 11 bp

Clone 2287 was transfected with the TALEN and ssODN followed by clone isolation as described for Figure 4. Genomic PCR of individual clones was carried out and the PCR products were TA cloned and sequenced. Part of the ssODN sequence is shown on top of the table and the homology regions between the ssODN and the wild-type allele are underlined. HDR denotes homology directed recombination. “Δ” denotes deletion which is indicated by a dished line.

## Discussion

In this study, we demonstrated the application of combining ssODN and TALEN to inactivate both of the miR-9-2 alleles in H9 cells. However, the efficiency of precise gene editing remains low (2 out of 300 H9 clones screened). In ESCs, the gene editing efficiency could be modulated by the TALEN cutting efficiency, the intracellular ssODN concentration and the accessibility of the genomic target. Using the GoldyTALEN scaffold, we showed enhanced target cutting which may be due to the additional truncations between the TALE repeats and the Fok1 nuclease domain in GoldyTALEN that increase the nuclease activity. We observed highly efficient NHEJ events at the miR-9-2 locus in the transfected H9 cells. Up to 17% of the transfected clones had NHEJ in the miR-9-2 locus, suggesting that the TALEN-mediated genomic cutting was not likely to be the rate-limiting step in gene editing. HDR-mediated gene editing also relies on donor template availability. We employed unmodified ssODN as the donor template. Single-stranded ODNs with phosphorothioate (PTO) modification were used to increase the stability of ssODNs [48]–[50]. However, PTO-protected ssODNs also increased the frequency of cell cycle arrest [51]. Incorporation of other types of nuclease-resistant residues such as 2′-O-methyl-ribonucleoside and locked nucleic acids in ssODN may avoid the adverse effects of PTO and increase the stability of ssODN [52]–[54].

Currently little information is available regarding TALEN target site accessibility. Bultmann *et al.* showed that 5-methylated cytosine in the genomic DNA had negative impact on TALE-mediated gene activation [55]. Chen *et al.* also observed a significant negative correlation between TALEN-mediated gene editing and the number of CpG repeats in TALEN target sites in zebrafish [56]. However, they found no such correlation in human cells. Our data does not support such a correlation either as some of our most efficient TALEN pairs have multiple CpG in their targets. Our results also showed that TALEN could cleave its genomic target irrespective of the gene activation status as TAT [57] and miR-9-2 genes (unpublished data) were silent in HEK293T cells, and their cutting efficiencies were comparable to actively expressed genes such as miR-21. Thus, target site accessibility could not account for the low gene editing efficiency in H9 cells. Homologous chromosome can also serve as template for DSB repair. Indeed, using clone 2287 containing heterozygous miR-9-2 alleles, we showed homologous chromosome-mediated HDR existed and the frequency was comparable to that of ssODN-mediated HDR. The involvement of homologous chromosome as a repair template for DSB may also explain the low efficiency of gaining the desired miR-9-2 deletion mutant as the wild-type allele in the homologous chromosome may compete against the transfected ssODN as a template to repair the DSB in the other allele.

Several groups have reported the highly efficient generation of germ-line transmittable gene knockouts in multiple species based on TALEN-mediated NHEJ events [22]–[28], [58]. However, small insertion or deletion generated by NHEJ in a gene may not be sufficient to completely abolish the encoded protein function. Precise deletion of multiple exons or even the entire gene may be preferable to ensure complete inactivation of the gene. Using two TALEN pairs in conjunction with an ssODN, we were able to generate the expected 7.8-kb deletion in all three TAT alleles in HEK293T cells at a frequency of 5.1%. This frequency was higher than the 0.3–1% reported previously by Lee *et al.* using two ZFN pairs without ssODN, and the 0.6% reported by Chen et al. using ssODN and one pair of ZFN [21], [59]. Our study therefore demonstrated the capacity of using TALEN and ssODN to create large genomic deletions efficiently. Such a strategy should also be applicable to other gene editing systems, such as CRISPR/Cas9.

The technologies for gene editing are fast evolving. In the recent two years, TALEN and CRISPR/Cas9 is well adopted due to the ease of nuclease assembly and high efficiency in gene editing. While CRISPR design is constrained by the requirement of the protospacer-adjacent motif (PAM) sequence (NGG) following the 20 bp CRISPR RNA target, no such restriction is known for TALENs. These two strategies are therefore complementary to each other for editing most of the genomic sequence. Currently one major concern of using these gene editing systems is the off-target effect on unintended genomic sequences. Off-target mutations can cause genome instability, DNA rearrangement and disruption of normal gene functions. The specificity of CRISPR relies on the 20 base guide sequence, whereas a TALEN pair targets approximately 30–40 bp genomic sequence. Recent studies showed that CRISPR/Cas9 could tolerate up to five mismatches between the guide RNA and its target to varying degree which confounded its application in research and therapeutics[10], [60], [61]. Although not immune to this problem, TALEN was reported to generate fewer off-target effects in general [13], [62]. However, to vigorously address this issue, an unbiased assay such as the use of integration defective lentiviral vectors to tag DSBs generated by TALEN will need to be employed [63].

In summary, our study shows that ssODN can serve as a feasible donor template for HDR in human cell lines including ESCs. Using ssODN in gene editing studies avoids the time-consuming step of constructing the template plasmid. It also avoids prolonged selection and removal of the introduced selectable marker from established ESC or iPSC clones. This novel gene editing technology should create a robust platform for dissecting genotype-phenotype relationships *in vitro*.

## Supporting Information

Figure S1 **Validation of TALEN cutting in HEK293T cells.** Each TALEN pair was transfected into HEK293T cells, and NHEJ was evaluated 48 h later with the Surveyor assay.
(TIF)

Figure S2 **Genotyping the miR-9-2 locus in the subclones derived from clone 2287.** Clone 2287 was treated with an additional round of gene knockout as described in Figure 4, and the genomic DNA from several subclones was isolated for PCR analysis. (A) Schematic drawing of the miR-9-2 locus and TALEN cutting site. The PCR primer pairs used for genotyping are shown. The two miR-9-2 alleles in clone 2287-19 and clone 2287-151 are also indicated. (B) PCR analysis of the genomic DNA isolated from 2287 subclones to detect miR-9-2 deletions. The primers used for this analysis are M9F2 and M9R2. The parental clone (clone 2287) is heterozygous for miR-9-2 with one wild-type allele and one deleted allele mediated by NHEJ. PCR amplification of the wild-type allele generates a 373 bp fragment and amplification of the deleted allele generates a 338 bp fragment. Knocking out the wild-type allele with ssODN generates a 284 bp fragment whereas knocking out the wild-type allele with the homologous chromosome generates two fragments with an identical size of 338 bp. Clones with ssODN-mediated HDR in the wild-type allele are indicated by “Δ”. The clone with homologous chromosome-mediated HDR is indicated by “*”. Both clone 2287-19 and clone 2287-151 exhibit only a single 338 bp PCR fragment. Additional PCR using M9F4 and M9R2 as primers showed that clone 2287-151 had an extended 235-bp deletion in the remaining wild-type allele (Table 3). This deletion, most likely generated by NHEJ, prevents the binding of M9F2 for PCR. (C) Verification of homozygous deletion in the miR-9-2 loci in clone 2287-19. M9F5 & R5, M9F6 & R6 were used in qPCR to measure the haploid copy number in regions flanking the TALEN cutting site in the miR-9-2 gene. The H9 genomic DNA was used as a calibrator. The GAPDH gene on chromosome 2 was used as a reference gene for gene copy normalization. PCR by both primer pairs shows that clone 2287-19 has an equivalent copy number as H9 in this genomic region. Due to NHEJ-induced 235 bp deletion, clone 2287-151 has only half of the copy as clone 2287-19 and H9 when M9F5 & R5 were used.
(TIF)

Table S1 **Sequence analysis of H9 clones with NHEJ.** Genomic PCR products from randomly isolated H9 clones transfected with the TALEN and ssODN were directly sequenced. The sequence was aligned with the sequence of ssODN and the wild-type miR-9-2 gene shown on top of the table. “Δ” denotes deletion and “I” denotes insertion. Inserted sequences are underlined and deletions are indicated by dashed lines.
(PDF)

Table S2 **Sequence analysis of H9 clones with large deletion in the miR-9-2 locus.** Genomic PCR products from the H9 clones with deletion in the miR-9-2 locus were TA cloned and sequenced. The sequence was aligned with the sequence of ssODN and the wild-type (WT) miR-9-2 gene shown on top of the table. “Δ” denotes deletion and “I” denotes insertion. The inserted sequence is underlined and deletions are indicated by dashed lines.
(PDF)

## References

[1] Urnov FD, Miller JC, Lee YL, Beausejour CM, Rock JM, et al (2005) Highly efficient endogenous human gene correction using designed zinc-finger nucleases. Nature 435: 646–651. 15806097 10.1038/nature03556

[2] Cong L, Ran FA, Cox D, Lin S, Barretto R, et al (2013) Multiplex genome engineering using CRISPR/Cas systems. Science 339: 819–823. 23287718 10.1126/science.1231143 PMC3795411

[3] Wang H, Yang H, Shivalila CS, Dawlaty MM, Cheng AW, et al (2013) One-step generation of mice carrying mutations in multiple genes by CRISPR/Cas-mediated genome engineering. Cell 153: 910–918. 23643243 10.1016/j.cell.2013.04.025 PMC3969854

[4] Ding Q, Lee YK, Schaefer EA, Peters DT, Veres A, et al (2013) A TALEN genome-editing system for generating human stem cell-based disease models. Cell Stem Cell 12: 238–251. 23246482 10.1016/j.stem.2012.11.011 PMC3570604

[5] Cermak T, Doyle EL, Christian M, Wang L, Zhang Y, et al (2011) Efficient design and assembly of custom TALEN and other TAL effector-based constructs for DNA targeting. Nucleic Acids Res 39: e82. 21493687 10.1093/nar/gkr218 PMC3130291

[6] Reyon D, Tsai SQ, Khayter C, Foden JA, Sander JD, et al (2012) FLASH assembly of TALENs for high-throughput genome editing. Nat Biotechnol 30: 460–465. 22484455 10.1038/nbt.2170 PMC3558947

[7] Mussolino C, Morbitzer R, Lutge F, Dannemann N, Lahaye T, et al (2011) A novel TALE nuclease scaffold enables high genome editing activity in combination with low toxicity. Nucleic Acids Res 39: 9283–9293. 21813459 10.1093/nar/gkr597 PMC3241638

[8] Hockemeyer D, Wang H, Kiani S, Lai CS, Gao Q, et al (2011) Genetic engineering of human pluripotent cells using TALE nucleases. Nat Biotechnol 29: 731–734. 21738127 10.1038/nbt.1927 PMC3152587

[9] Hsu PD, Scott DA, Weinstein JA, Ran FA, Konermann S, et al (2013) DNA targeting specificity of RNA-guided Cas9 nucleases. Nat Biotechnol 31: 827–832. 23873081 10.1038/nbt.2647 PMC3969858

[10] Fu Y, Foden JA, Khayter C, Maeder ML, Reyon D, et al (2013) High-frequency off-target mutagenesis induced by CRISPR-Cas nucleases in human cells. Nat Biotechnol 31: 822–826. 23792628 10.1038/nbt.2623 PMC3773023

[11] Cho SW, Kim S, Kim Y, Kweon J, Kim HS, et al.. (2013) Analysis of off-target effects of CRISPR/Cas-derived RNA-guided endonucleases and nickases. Genome Res. 10.1101/gr.162339.113 PMC3875854 24253446

[12] Sanjana NE, Cong L, Zhou Y, Cunniff MM, Feng G, et al (2012) A transcription activator-like effector toolbox for genome engineering. Nat Protoc 7: 171–192. 22222791 10.1038/nprot.2011.431 PMC3684555

[13] Osborn MJ, Starker CG, McElroy AN, Webber BR, Riddle MJ, et al (2013) TALEN-based gene correction for epidermolysis bullosa. Mol Ther 21: 1151–1159. 23546300 10.1038/mt.2013.56 PMC3677309

[14] Li T, Huang S, Zhao X, Wright DA, Carpenter S, et al (2011) Modularly assembled designer TAL effector nucleases for targeted gene knockout and gene replacement in eukaryotes. Nucleic Acids Res 39: 6315–6325. 21459844 10.1093/nar/gkr188 PMC3152341

[15] Christian M, Cermak T, Doyle EL, Schmidt C, Zhang F, et al (2010) Targeting DNA double-strand breaks with TAL effector nucleases. Genetics 186: 757–761. 20660643 10.1534/genetics.110.120717 PMC2942870

[16] Mak AN, Bradley P, Cernadas RA, Bogdanove AJ, Stoddard BL (2012) The crystal structure of TAL effector PthXo1 bound to its DNA target. Science 335: 716–719. 22223736 10.1126/science.1216211 PMC3427646

[17] Deng D, Yan C, Pan X, Mahfouz M, Wang J, et al (2012) Structural basis for sequence-specific recognition of DNA by TAL effectors. Science 335: 720–723. 22223738 10.1126/science.1215670 PMC3586824

[18] Streubel J, Blucher C, Landgraf A, Boch J (2012) TAL effector RVD specificities and efficiencies. Nat Biotechnol 30: 593–595. 22781676 10.1038/nbt.2304

[19] Li T, Huang S, Jiang WZ, Wright D, Spalding MH, et al (2010) TAL nucleases (TALNs): hybrid proteins composed of TAL effectors and FokI DNA-cleavage domain. Nucleic Acids Res 39: 359–372. 20699274 10.1093/nar/gkq704 PMC3017587

[20] Mahfouz MM, Li L, Shamimuzzaman M, Wibowo A, Fang X, et al (2011) De novo-engineered transcription activator-like effector (TALE) hybrid nuclease with novel DNA binding specificity creates double-strand breaks. Proc Natl Acad Sci U S A 108: 2623–2628. 21262818 10.1073/pnas.1019533108 PMC3038751

[21] Chen F, Pruett-Miller SM, Huang Y, Gjoka M, Duda K, et al (2011) High-frequency genome editing using ssDNA oligonucleotides with zinc-finger nucleases. Nat Methods 8: 753–755. 21765410 10.1038/nmeth.1653 PMC3617923

[22] Bedell VM, Wang Y, Campbell JM, Poshusta TL, Starker CG, et al (2012) In vivo genome editing using a high-efficiency TALEN system. Nature 491: 114–118. 23000899 10.1038/nature11537 PMC3491146

[23] Carlson DF, Tan W, Lillico SG, Stverakova D, Proudfoot C, et al (2012) Efficient TALEN-mediated gene knockout in livestock. Proc Natl Acad Sci U S A 109: 17382–17387. 23027955 10.1073/pnas.1211446109 PMC3491456

[24] Huang P, Xiao A, Zhou M, Zhu Z, Lin S, et al (2011) Heritable gene targeting in zebrafish using customized TALENs. Nat Biotechnol 29: 699–700. 21822242 10.1038/nbt.1939

[25] Sander JD, Cade L, Khayter C, Reyon D, Peterson RT, et al (2011) Targeted gene disruption in somatic zebrafish cells using engineered TALENs. Nat Biotechnol 29: 697–698. 21822241 10.1038/nbt.1934 PMC3154023

[26] Tong C, Huang G, Ashton C, Wu H, Yan H, et al (2012) Rapid and cost-effective gene targeting in rat embryonic stem cells by TALENs. J Genet Genomics 39: 275–280. 22749015 10.1016/j.jgg.2012.04.004 PMC3856761

[27] Wefers B, Meyer M, Ortiz O, Hrabe de Angelis M, Hansen J, et al (2013) Direct production of mouse disease models by embryo microinjection of TALENs and oligodeoxynucleotides. Proc Natl Acad Sci U S A 110: 3782–3787. 23426636 10.1073/pnas.1218721110 PMC3593923

[28] Lei Y, Guo X, Liu Y, Cao Y, Deng Y, et al (2012) Efficient targeted gene disruption in Xenopus embryos using engineered transcription activator-like effector nucleases (TALENs). Proc Natl Acad Sci U S A 109: 17484–17489. 23045671 10.1073/pnas.1215421109 PMC3491516

[29] Hu R, Wallace J, Dahlem TJ, Grunwald DJ, O'Connell RM (2013) Targeting human microRNA genes using engineered Tal-effector nucleases (TALENs). PLoS One 8: e63074. 23667577 10.1371/journal.pone.0063074 PMC3646762

[30] Takada S, Sato T, Ito Y, Yamashita S, Kato T, et al (2013) Targeted Gene Deletion of miRNAs in Mice by TALEN System. PLoS One 8: e76004. 24146809 10.1371/journal.pone.0076004 PMC3797721

[31] Kim YK, Wee G, Park J, Kim J, Baek D, et al.. (2013) TALEN-based knockout library for human microRNAs. Nat Struct Mol Biol. 10.1038/nsmb.2701 24213537

[32] Moynahan ME, Jasin M (2010) Mitotic homologous recombination maintains genomic stability and suppresses tumorigenesis. Nat Rev Mol Cell Biol 11: 196–207. 20177395 10.1038/nrm2851 PMC3261768

[33] Doyle EL, Booher NJ, Standage DS, Voytas DF, Brendel VP, et al (2012) TAL Effector-Nucleotide Targeter (TALE-NT) 2.0: tools for TAL effector design and target prediction. Nucleic Acids Res 40: W117–122. 22693217 10.1093/nar/gks608 PMC3394250

[34] Hu BY, Weick JP, Yu J, Ma LX, Zhang XQ, et al (2010) Neural differentiation of human induced pluripotent stem cells follows developmental principles but with variable potency. Proc Natl Acad Sci U S A 107: 4335–4340. 20160098 10.1073/pnas.0910012107 PMC2840097

[35] De Preter K, Speleman F, Combaret V, Lunec J, Laureys G, et al (2002) Quantification of MYCN, DDX1, and NAG gene copy number in neuroblastoma using a real-time quantitative PCR assay. Mod Pathol 15: 159–166. 11850545 10.1038/modpathol.3880508

[36] Boch J, Scholze H, Schornack S, Landgraf A, Hahn S, et al (2009) Breaking the code of DNA binding specificity of TAL-type III effectors. Science 326: 1509–1512. 19933107 10.1126/science.1178811

[37] Moscou MJ, Bogdanove AJ (2009) A simple cipher governs DNA recognition by TAL effectors. Science 326: 1501. 19933106 10.1126/science.1178817

[38] Shibata M, Kurokawa D, Nakao H, Ohmura T, Aizawa S (2008) MicroRNA-9 modulates Cajal-Retzius cell differentiation by suppressing Foxg1 expression in mouse medial pallium. J Neurosci 28: 10415–10421. 18842901 10.1523/JNEUROSCI.3219-08.2008 PMC6671033

[39] Leucht C, Stigloher C, Wizenmann A, Klafke R, Folchert A, et al (2008) MicroRNA-9 directs late organizer activity of the midbrain-hindbrain boundary. Nat Neurosci 11: 641–648. 18454145 10.1038/nn.2115

[40] Uchida N (2010) MicroRNA-9 controls a migratory mechanism in human neural progenitor cells. Cell Stem Cell 6: 294–296. 20362531 10.1016/j.stem.2010.03.010

[41] Dajas-Bailador F, Bonev B, Garcez P, Stanley P, Guillemot F, et al (2012) microRNA-9 regulates axon extension and branching by targeting Map1b in mouse cortical neurons. Nat Neurosci 15: 697–699. 10.1038/nn.3082 22484572

[42] Delaloy C, Liu L, Lee JA, Su H, Shen F, et al (2010) MicroRNA-9 coordinates proliferation and migration of human embryonic stem cell-derived neural progenitors. Cell Stem Cell 6: 323–335. 20362537 10.1016/j.stem.2010.02.015 PMC2851637

[43] Yuva-Aydemir Y, Simkin A, Gascon E, Gao FB (2011) MicroRNA-9: functional evolution of a conserved small regulatory RNA. RNA Biol 8: 557–564. 21697652 10.4161/rna.8.4.16019 PMC3225974

[44] Shibata M, Nakao H, Kiyonari H, Abe T, Aizawa S (2011) MicroRNA-9 regulates neurogenesis in mouse telencephalon by targeting multiple transcription factors. J Neurosci 31: 3407–3422. 21368052 10.1523/JNEUROSCI.5085-10.2011 PMC6623912

[45] Laneve P, Gioia U, Andriotto A, Moretti F, Bozzoni I, et al (2010) A minicircuitry involving REST and CREB controls miR-9-2 expression during human neuronal differentiation. Nucleic Acids Res 38: 6895–6905. 20624818 10.1093/nar/gkq604 PMC2978373

[46] Gandhi M, Evdokimova VN, K TC, Nikiforova MN, Kelly LM, et al (2012) Homologous chromosomes make contact at the sites of double-strand breaks in genes in somatic G0/G1-phase human cells. Proc Natl Acad Sci U S A 109: 9454–9459. 22645362 10.1073/pnas.1205759109 PMC3386068

[47] Richardson C, Moynahan ME, Jasin M (1998) Double-strand break repair by interchromosomal recombination: suppression of chromosomal translocations. Genes Dev 12: 3831–3842. 9869637 10.1101/gad.12.24.3831 PMC317271

[48] Campbell CR, Keown W, Lowe L, Kirschling D, Kucherlapati R (1989) Homologous recombination involving small single-stranded oligonucleotides in human cells. New Biol 1: 223–227. 2562222

[49] Radecke S, Radecke F, Peter I, Schwarz K (2006) Physical incorporation of a single-stranded oligodeoxynucleotide during targeted repair of a human chromosomal locus. J Gene Med 8: 217–228. 16142817 10.1002/jgm.828

[50] Papaioannou I, Disterer P, Owen JS (2009) Use of internally nuclease-protected single-strand DNA oligonucleotides and silencing of the mismatch repair protein, MSH2, enhances the replication of corrected cells following gene editing. J Gene Med 11: 267–274. 19153972 10.1002/jgm.1296

[51] Aarts M, te Riele H (2010) Subtle gene modification in mouse ES cells: evidence for incorporation of unmodified oligonucleotides without induction of DNA damage. Nucleic Acids Res 38: 6956–6967. 20601408 10.1093/nar/gkq589 PMC2978364

[52] Igoucheva O, Alexeev V, Anni H, Rubin E (2008) Oligonucleotide-mediated gene targeting in human hepatocytes: implications of mismatch repair. Oligonucleotides 18: 111–122. 18637729 10.1089/oli.2008.0120 PMC2966837

[53] Igoucheva O, Alexeev V, Scharer O, Yoon K (2006) Involvement of ERCC1/XPF and XPG in oligodeoxynucleotide-directed gene modification. Oligonucleotides 16: 94–104. 16584298 10.1089/oli.2006.16.94

[54] Andrieu-Soler C, Casas M, Faussat AM, Gandolphe C, Doat M, et al (2005) Stable transmission of targeted gene modification using single-stranded oligonucleotides with flanking LNAs. Nucleic Acids Res 33: 3733–3742. 16002788 10.1093/nar/gki686 PMC1174897

[55] Bultmann S, Morbitzer R, Schmidt CS, Thanisch K, Spada F, et al (2012) Targeted transcriptional activation of silent oct4 pluripotency gene by combining designer TALEs and inhibition of epigenetic modifiers. Nucleic Acids Res 40: 5368–5377. 22387464 10.1093/nar/gks199 PMC3384321

[56] Chen S, Oikonomou G, Chiu CN, Niles BJ, Liu J, et al (2013) A large-scale in vivo analysis reveals that TALENs are significantly more mutagenic than ZFNs generated using context-dependent assembly. Nucleic Acids Res 41: 2769–2778. 23303782 10.1093/nar/gks1356 PMC3575824

[57] Dvorak Z, Modriansky M, Pichard-Garcia L, Balaguer P, Vilarem MJ, et al (2003) Colchicine down-regulates cytochrome P450 2B6, 2C8, 2C9, and 3A4 in human hepatocytes by affecting their glucocorticoid receptor-mediated regulation. Mol Pharmacol 64: 160–169. 12815172 10.1124/mol.64.1.160

[58] Ding Q, Lee YK, Schaefer EA, Peters DT, Veres A, et al (2013) A TALEN genome-editing system for generating human stem cell-based disease models. Cell Stem Cell 12: 238–251. 23246482 10.1016/j.stem.2012.11.011 PMC3570604

[59] Lee HJ, Kim E, Kim JS (2010) Targeted chromosomal deletions in human cells using zinc finger nucleases. Genome Res 20: 81–89. 19952142 10.1101/gr.099747.109 PMC2798833

[60] Cradick TJ, Fine EJ, Antico CJ, Bao G (2013) CRISPR/Cas9 systems targeting beta-globin and CCR5 genes have substantial off-target activity. Nucleic Acids Res 41: 9584–9592. 23939622 10.1093/nar/gkt714 PMC3814385

[61] Cho SW, Kim S, Kim Y, Kweon J, Kim HS, et al (2014) Analysis of off-target effects of CRISPR/Cas-derived RNA-guided endonucleases and nickases. Genome Res 24: 132–141. 24253446 10.1101/gr.162339.113 PMC3875854

[62] Fine EJ, Cradick TJ, Zhao CL, Lin Y, Bao G (2013) An online bioinformatics tool predicts zinc finger and TALE nuclease off-target cleavage. Nucleic Acids Res. 10.1093/nar/gkt1326 PMC3973315 24381193

[63] Gabriel R, Lombardo A, Arens A, Miller JC, Genovese P, et al (2011) An unbiased genome-wide analysis of zinc-finger nuclease specificity. Nat Biotechnol 29: 816–823. 21822255 10.1038/nbt.1948

