# Supplementary figures and images for: Precise Gene Modification Mediated by TALEN and Single-Stranded Oligodeoxynucleotides in Human Cells
Source: PLoS One. 2014 Apr 1;9(4):e93575. doi: 10.1371/journal.pone.0093575 (PMC3972112; doi:10.1371/journal.pone.0093575)

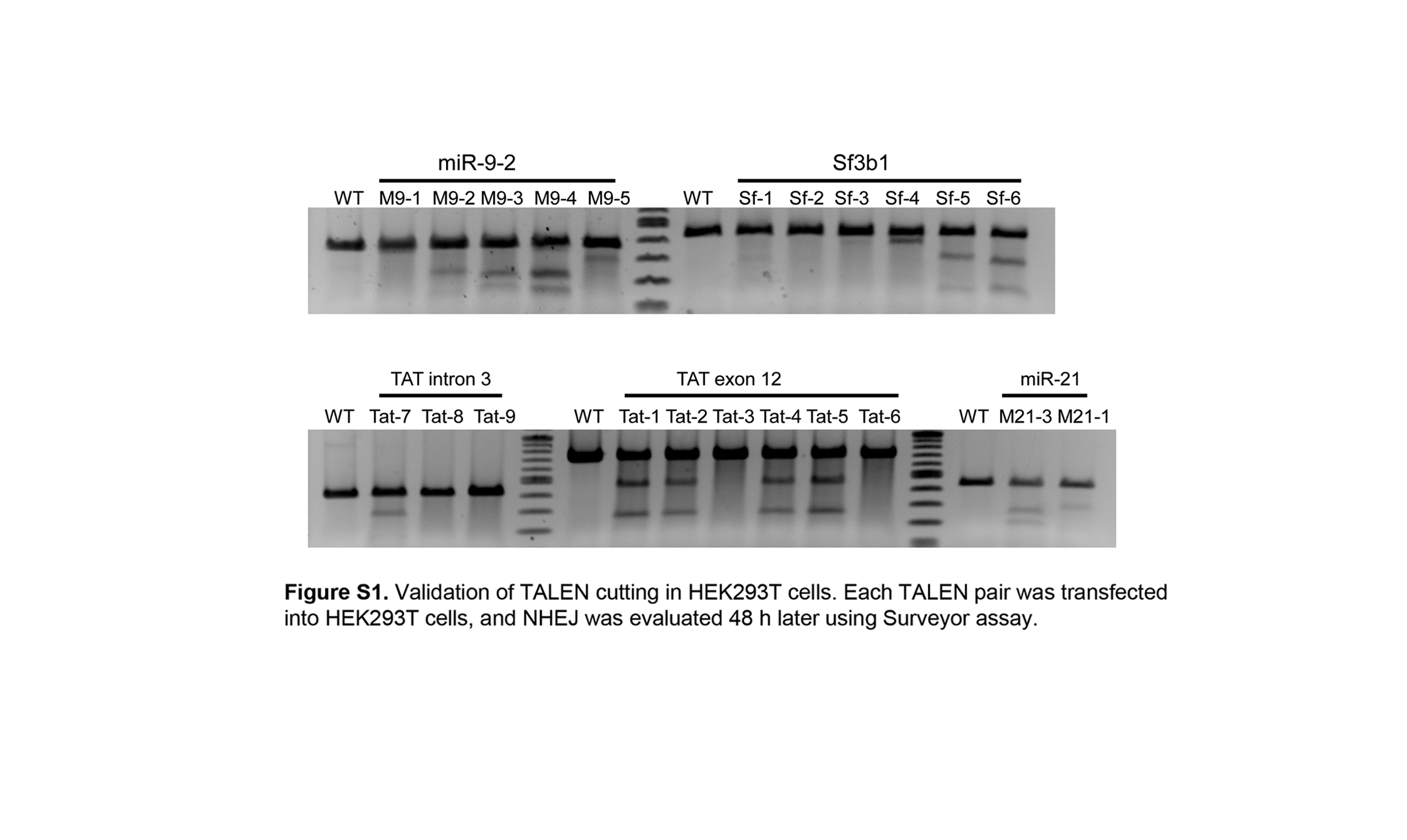

Supplement: Figure S1 — Validation of TALEN cutting in HEK293T cells. Each TALEN pair was transfected into HEK293T cells, and NHEJ was evaluated 48 h later with the Surveyor assay. (TIF) [file pone.0093575.s001.tif]

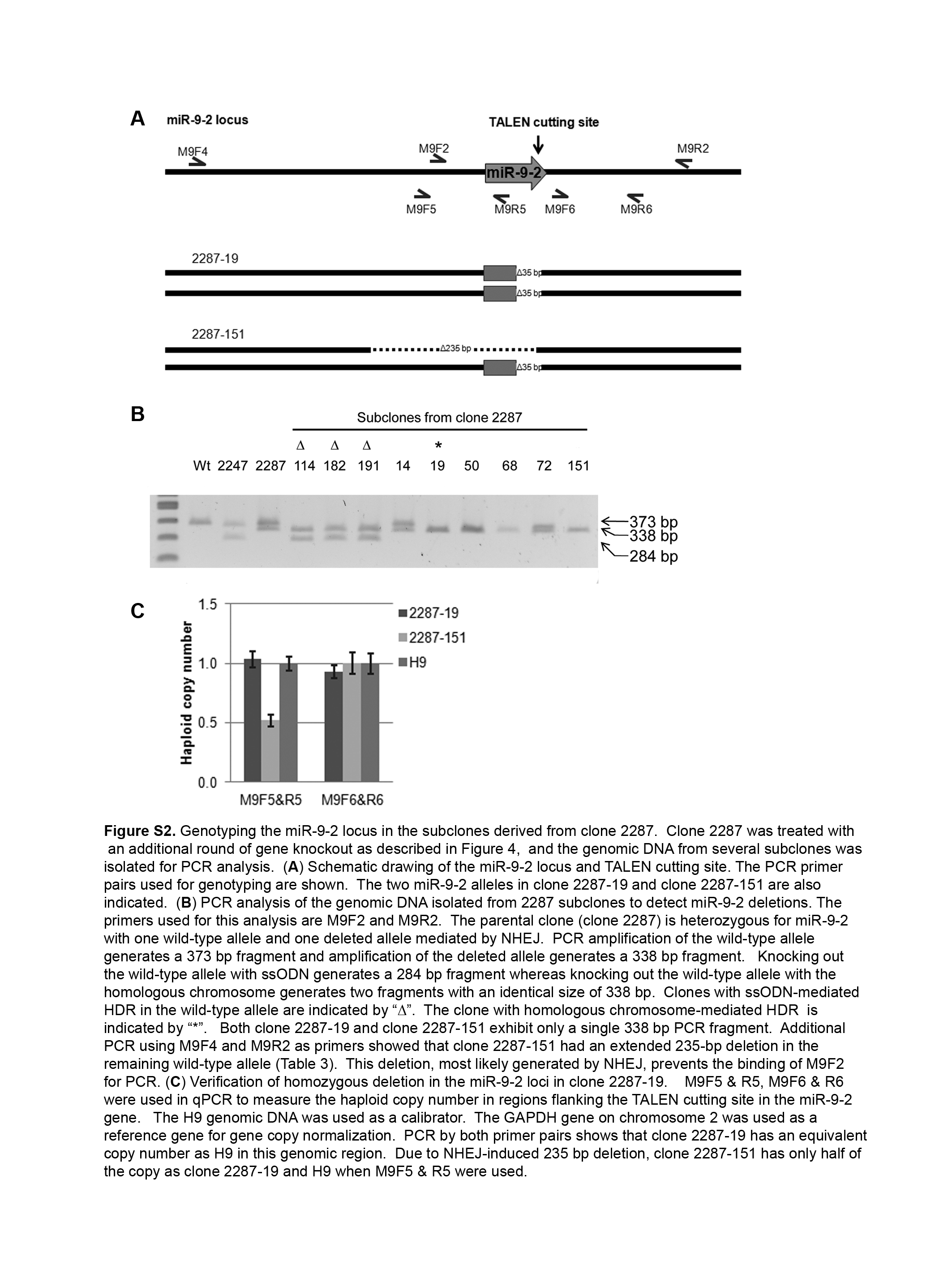

Supplement: Figure S2 — Genotyping the miR-9-2 locus in the subclones derived from clone 2287. Clone 2287 was treated with an additional round of gene knockout as described in Figure 4, and the genomic DNA from several subclones was isolated for PCR analysis. (A) Schematic drawing of the miR-9-2 locus and TALEN cutting site. The PCR primer pairs used for genotyping are shown. The two miR-9-2 alleles in clone 2287-19 and clone 2287-151 are also indicated. (B) PCR analysis of the genomic DNA isolated from 2287 subclones to detect miR-9-2 deletions. The primers used for this analysis are M9F2 and M9R2. The parental clone (clone 2287) is heterozygous for miR-9-2 with one wild-type allele and one deleted allele mediated by NHEJ. PCR amplification of the wild-type allele generates a 373 bp fragment and amplification of the deleted allele generates a 338 bp fragment. Knocking out the wild-type allele with ssODN generates a 284 bp fragment whereas knocking out the wild-type allele with the homologous chromosome generates two fragments with an identical size of 338 bp. Clones with ssODN-mediated HDR in the wild-type allele are indicated by “Δ”. The clone with homologous chromosome-mediated HDR is indicated by “*”. Both clone 2287-19 and clone 2287-151 exhibit only a single 338 bp PCR fragment. Additional PCR using M9F4 and M9R2 as primers showed that clone 2287-151 had an extended 235-bp deletion in the remaining wild-type allele (Table 3). This deletion, most likely generated by NHEJ, prevents the binding of M9F2 for PCR. (C) Verification of homozygous deletion in the miR-9-2 loci in clone 2287-19. M9F5 & R5, M9F6 & R6 were used in qPCR to measure the haploid copy number in regions flanking the TALEN cutting site in the miR-9-2 gene. The H9 genomic DNA was used as a calibrator. The GAPDH gene on chromosome 2 was used as a reference gene for gene copy normalization. PCR by both primer pairs shows that clone 2287-19 has an equivalent copy number as H9 in this genomic region. Due to NHEJ-induced 235 [file pone.0093575.s002.tif]
