# Supplementary material for: Precise Gene Modification Mediated by TALEN and Single-Stranded Oligodeoxynucleotides in Human Cells
Source: PLoS One. 2014 Apr 1;9(4):e93575. doi: 10.1371/journal.pone.0093575 (PMC3972112; doi:10.1371/journal.pone.0093575)
Supplement: Table S1 — Sequence analysis of H9 clones with NHEJ. Genomic PCR products from randomly isolated H9 clones transfected with the TALEN and ssODN were directly sequenced. The sequence was aligned with the sequence of ssODN and the wild-type miR-9-2 gene shown on top of the table. “Δ” denotes deletion and “I” denotes insertion. Inserted sequences are underlined and deletions are indicated by dashed lines. (PDF) [file pone.0093575.s003.pdf]

Table S1. Sequence analysis of H9 clones with NHEJ.

	Sequence	Genotype
	ACAGAGG.65bp. GCTAGATAACCGAAAGTAAAAACTCCTTCAAGATCGCCGG	WT
	ACAGAGGaTCCTTCAAGATCGCCGG	ssODN
clone 2107	ACAGAGG.65bp. GCTAGATAACCG-------AAACTCCTTCAAGATCGCCGG	Δ 7 bp
	ACAGAGG.39bp. --------------AGTAAAAACTCCTTCAAGATCGCCGG	Δ 40 bp
clone 2106	ACAGAGG.65bp. GCTAGATAACCGAAAGTAAAAAACTCCTTCAAGATCGCCGG	I 1 bp
	ACAGAGG.47bp. ----------------------CTCCTTCAAGATCGCCGG	Δ 40 bp
clone 2129	ACAGAGG.65bp. GCTAGATAACCG-------AAACTCCTTCAAGATCGCCGG	Δ 7 bp
	ACAGAGG.65bp. GCTAGATAACCGAAAGT----------TCAAGATCGCCGG	Δ 10 bp
clone 2143	ACAGAGG.65bp. GCTAG-------------AAACTCCTTCAAGATCGCCGG	Δ 13 bp
	ACAGAGG.65bp. GCTAGATAACCG-AAGTAAAAACTCCTTCAAGATCGCCGG	Δ 1 bp
clone 2170	ACAGAGG.65bp. GCTAGATAACCGAAAGT-AAAACTCCTTCAAGATCGCCGG	Δ 1 bp
	ACAGAGG.65bp. GCT-----------AGTAAAAACTCCTTCAAGATCGCCGG	Δ 11 bp
clone 2101	ACAGAGG.65bp. GCTAGATAACCGA----AAAAACTCCTTCAAGATCGCCGG	Δ 4 bp
	ACAGAGG.65bp. GCTAGATAACCGAAAGTAAAAACTCCTTCAAGATCGCCGG	WT
clone 2144	ACAGAGG.65bp. GCTAGATAACCGAAAGTAAAAACTCCTTCAAGATCGCCGG	WT
	ACAGAGG.65bp. GCTAGATAACCGAAAGTTAAAAACTCCTTCAAGATCGCCGG	I 1 bp
clone 2148	ACAGAGG.65bp. GCTAGATAACCGAAAGTAAAAACTCCTTCAAGATCGCCGG	WT
	ACAGAGG.65bp. GCTAGATAACCGAAAAGTAAAAACTCCTTCAAGATCGCCGG	I 1 bp
clone 2152	ACAGAGG.65bp. GCTAGATAACCGAAAGTAAAAACTCCTTCAAGATCGCCGG	WT
	ACAGAGG.65bp. GCTAGATAACCGAAAGTTAAAAACTCCTTCAAGATCGCCGG	I 1 bp
clone 2157	ACAGAGG.65bp. GCTAGATAACCGAAAGTAAAAACTCCTTCAAGATCGCCGG	WT
	ACAGAGG.65bp. GCTAGATAACCGAAAGT-AAAACTCCTTCAAGATCGCCGG	Δ 1 bp
clone 2158	ACAGAGG.65bp. GCTAGATAACCGAAAGTAAAAACTCCTTCAAGATCGCCGG	WT
	ACAGAGG.65bp. GCTAGATAACCGAAAGTTAAAAACTCCTTCAAGATCGCCGG	I 1 bp
clone 2162	ACAGAGG.65bp. GCTAGATAACCGAAAGTAAAAACTCCTTCAAGATCGCCGG	WT
	ACAGAGG.65bp. GCTAGATAACCG-AAGTAAAAACTCCTTCAAGATCGCCGG	Δ 1 bp
clone 2164	ACAGAGG.65bp. GCTAGATAACCGAAAGTAAAAACTCCTTCAAGATCGCCGG	WT
	ACAGAGG.65bp. GCTAGATAACCGAAA----------------GATCGCCGG	Δ 15 bp
clone 2175	ACAGAGG.65bp. GCTAGATAACCGAAAGTAAAAACTCCTTCAAGATCGCCGG	WT
	ACAGAGG.65bp. GCTAGATAACCGA----AAAAACTCCTTCAAGATCGCCGG	Δ 4 bp
clone 2185	ACAGAGG.65bp. GCTAGATAACCGAAAGTAAAAACTCCTTCAAGATCGCCGG	WT
	ACAGAGG.65bp. GCTAGATAACCGAA--TAAAAACTCCTTCAAGATCGCCGG	Δ 2 bp

Genomic PCR products from randomly isolated H9 clones transfected with the TALEN and ssODN were directly sequenced. The sequence was aligned with the sequence of ssODN and the wild-type miR-9-2 gene shown on top of the table. “Δ” denotes deletion and “I” denotes insertion. Inserted sequences are underlined and deletions are indicated by dashed lines.
