# Supplementary material for: Precise Gene Modification Mediated by TALEN and Single-Stranded Oligodeoxynucleotides in Human Cells
Source: PLoS One. 2014 Apr 1;9(4):e93575. doi: 10.1371/journal.pone.0093575 (PMC3972112; doi:10.1371/journal.pone.0093575)
Supplement: Table S2 — Sequence analysis of H9 clones with large deletion in the miR-9-2 locus. Genomic PCR products from the H9 clones with deletion in the miR-9-2 locus were TA cloned and sequenced. The sequence was aligned with the sequence of ssODN and the wild-type (WT) miR-9-2 gene shown on top of the table. “Δ” denotes deletion and “I” denotes insertion. The inserted sequence is underlined and deletions are indicated by dashed lines. (PDF) [file pone.0093575.s004.pdf]

Table S2. Sequence analysis of H9 clones with large deletion in the miR-9-2 locus.

	Sequence	Genotype
	ACAGAGG.65bp. GCTAGATAACCGAAAGTAAAAACTCCTTCAAGATCGCCGG<br>ACAGAGGaTCCTTCAAGATCGCCGG	WT<br>HDR
clone 2079	ACAGAGG.65bp. GCTAGATAACCGAAAGTTAAAAACTCCTTCAAGATCGCCGG<br>ACAGAGGaTCCTTCAAGATCGCCGG	I 1 bp<br>HDR
clone 2247	ACAGAGGaTCCTTCAAGATCGCCGG<br>ACAGAGG.65bp. GCTAG-------------AAAACTCCTTCAAGATCGCCGG	HDR<br>Δ 13 bp
clone 2287	ACAGAGG.65bp. GCTAGATAACCGAAAGTAAAAACTCCTTCAAGATCGCCGG<br>ACAGAGG.55bp. -------------------------CTTCAAGATCGCCGG	WT<br>Δ 35 bp

Genomic PCR products from the H9 clones with deletion in the miR-9-2 locus were TA cloned and sequenced. The sequence was aligned with the sequence of ssODN and the wild-type (WT) miR-9-2 gene shown on top of the table. “Δ” denotes deletion and “I” denotes insertion. The inserted sequence is underlined and deletions are indicated by dashed lines.
